# Translation and validation of Suicide Risk Scale for Medical Inpatient (SRMI) in Urdu Language

**DOI:** 10.12669/pjms.39.6.7000

**Published:** 2023

**Authors:** Mobeen Mehmood, Noshi Iram Zaman

**Affiliations:** 1 Mobeen Mehmood MS Scholar, Department of Professional Psychology, Bahria University, Islamabad, Pakistan; 2 Dr. Noshi Iram Zaman Assistant Professor, Department of Professional Psychology, Bahria University, Islamabad, Pakistan

**Keywords:** Suicidal risk, Translation, Validation, Renal Failure patients

## Abstract

**Objective:**

To translate and validate the suicide risk scale for medical inpatients in the Urdu language.

**Methods:**

A correlational study with non-random sample approach was conducted from February to May 2020, data was collected from various hospitals in Rawalpindi, Islamabad, and Lahore. In the first phase, the suicide risk scale for medical inpatients was translated from the source language (English) to the target language (Urdu) via forward and backward translations. In the second phase, the Psychometric properties of the Urdu version were established. Exploratory factor analysis, reliability (Cronbach’s alpha & split-half), and validity analysis (convergent and discriminant) were also computed.

**Results:**

A total of 200 individuals with renal failure, with a mean age of 45.33 years (range; 18-80 years) were approached for the data collection. Exploratory factor analysis computed two factors. Cronbach’s alpha value showed internal consistency and Pearson’s moment correlation indicated association among similar variables (convergent validity) and less association among dissimilar study variables (discriminant validity).

**Conclusion:**

The Urdu version of the suicide risk scale for medical inpatients was found to be a reliable and valid instrument for assessing suicide risk in medical inpatients in Pakistani culture.

## INTRODUCTION

Suicide is a major public health concern and the leading death cause across various age groups globally.^1^ It is expected that between 0.97% and 1.9% of suicides take place in general hospitals.^2^ According to an estimated account five to fifteen patients deaths per 100,000 admissions in general hospitals.^3^ Evidence showed that 75% of suicides occurred in low-income and middle-income countries.^4^ Countries like Pakistan, have weak health systems with scanty health resources^5^, and a shocking increase in suicidal behavior is significantly alarming. According to the WHO report, in Pakistan, 7000-8000 people commit suicide, 70,000-150,000 people try to commit suicide and many of them remained unreported.^6^ Suicide can be defined as a self-injurious behavior with a wish to die.^7^ Poor physical health is a well-known risk factor for suicide because it affects the quality-of-life negatively.^8^ The literature has found a link between physical illness and suicide. The physical illness causes great pain which may take away the patient’s will to live and increase the risk of suicide.^9^ Various physical diseases make patients more prone to suicide, for example, cancer, coronary heart disease, CNS dysfunction, and renal failure are expected to commit suicide in contrast to the general population.^10^

A significant positive association between inward anger and suicidal ideation among hemodialysis patients was found.^11^ Moreover, patients with low HB levels and hyponatremia have suicidal ideation.^12^ Other factors that are associated with high suicide risk are chronic pain, social isolation, hopelessness older age, financial problems, reduce movement, and feelings of burdensomeness all these factor uplift suicide risk. Evidence suggested that functional limitations due to chronic illness are one of the strong predictors of suicide.^10^-^13^ Studies have showed, women frequently attempt suicide as compared to men whereas, death as a result of suicide is more common in men.^14^ Women as compared to men are more prone to physical health problems that contribute to suicide before the age of 60 years. Elder men are more prone to physical illness and consequently towards suicide.^15^ Research showed that almost 95% of individuals who attempted suicide visited medical care a year before their death.^16^ Forty five percent of individuals visit the hospital within a month before committing suicide without telling regarding their plan of suicide and 16% of individuals who commit suicide once may try a second attempt within a year.^17^

Studies have suggested that communication gaps and difficulties are one factor behind suicide. When a person feels alone and unable to share mental pain, it will lead to negative thoughts, and reduce self-worth, consequently they commit suicide.^18^ Whether depression and suicidal ideation are untreated for a long time, may cause poor quality of life even leading to death.^19^ The health care providers have a crucial role in identifying the patients who are at risk, thus they have to be vigilant.^20^ Inpatient suicide is one of the most important issues regarding hospital safety. So, these patients were kept on constant medical observation by the medical staff to observe the possible warning signs. But unfortunately, Inpatients do commit suicide during the supervision of medical staff because their risk assessment and prevention have not been done timely. The medical staff feels sad and guilty because they consider it their incompetence.^21^ A suicide that happens in a medical setting differs in terms of unique characteristics compared to a suicide that occurs in a psychiatric setting. In the psychiatric unit consultants and staff keep a close observation of high-risk patients, and assess possible warning signs as well as behavioral changes. They use various reliable assessment tools to assess suicide risk and mental examination from time to time and provide interventions.^22^ Whereas, in a medical setting physicians and staff focus on patients’ medical conditions and they don’t have time for detailed assessment and observation. Therefore, they need culture-fair short measures for the assessment of possible suicide risk in medical inpatients. So, they can provide safe and a favorable hospital environment.^23^ Initial assessment and intervention can act as a protective approach against suicidal behavior. Therefore, healthcare providers must monitor patients carefully and frequently, educate patients about issues with their families, and warn them not to keep medications or other harmful stuff close to them.^24^ It is essential that healthcare providers completely assess suicide risk and take realistic steps to assure the protection of a suicidal patient. It is crucial to use valid, culture-fair, and population-specific instruments for screening. Though there are few valid screening tools for medical patients.^25^ Thus, the purpose of the current study was to translate and validate a reliable screening tool that can quickly assess suicide risk in a medical setting.

Suicide risk for medical inpatients (SRMI) was developed by Park, et al, who used to assess suicide risk. It is a four-point Likert scale and contains seven items. Scores range from 0 to 3 (0; strongly disagree, 1; disagree, 2; agree, and 3; strongly agree). The cut-off score is five. SRMI has high internal consistency i.e., 0.91. This scale was originally developed in the Korean language and then translated into the English language.^26^

## METHODS

The Correlational study was conducted at various hospitals in Rawalpindi, Islamabad, and Lahore from February to May 2020.

### Ethical Approval:

The Ethical review committee of Bahria University Islamabad approved the study with a letter number. Ref. No: BUIC/HoD PP/2019/021 on 22 October 2021. The research project is according to the Ethical Standards of International Research and is the most needed domain in the social sciences fraternity.

In the first phase of the study translation of the scale (SRMI) into the Urdu language was done as per guidelines of the World Health Organization (2016).^27^ In forward translation, scale was translated from the English language to the Urdu language. Four bilingual persons who are experts in understanding both languages (English and Urdu) as well as acquainted with the terminology of the area covered by the instrument were approached for forward translation. The translators were instructed to translate the scale into simple language so that participants can comprehend the language without any hurdles. All four translators were contacted independently. Important information regarding the translation procedure was communicated to experts and requested to not omit any item. The committee consisted of four professionals, subject matter experts, and bilinguals (proficient in both Urdu and English languages). They evaluated each item in terms of accuracy, semantics, idiomatic, and conveying the meaning of the original items. Discrepancies between forward translation and original existing versions of questions were also checked. The assessment and selection of translation were based on simple language, understandable, and sentence structure according to cultural context done by the committee. The most suitable translation of each item close to the foundation language (English) was carefully selected. In backward translation, scale is translated back to the original language to assess the accuracy and quality of translation. For this purpose, another independent bilingual expert panel was approached. They checked the conceptual equivalence of translated version compared to the original one. The pre-final version of the translated scale was pilot tested on 50 individuals with renal failure who can read the Urdu language. They didn’t show any difficulty while responding on the scale. The researcher made sure to check for any difficulty in understanding the items. Participants didn’t report any confusion regarding statements on the questionnaire. Thus, the final version of the Suicide Risk Scale for Medical inpatients (SRMI) was achieved and administered to 200 individuals with renal failure.

### Statistical Analysis:

SPSS V 25 was used for the calculation of the results. Internal consistency within a scale was assessed through Cronbach’s alpha. Kaiser-Meyer-Olkin’s (KMO) and Bartlett’s test of sphericity were used to examine the sampling adequacy and data reduction. Pearson’s movement of correlation was used for the estimation of the validity of the scale.

## RESULTS

There were 200 individuals with renal failure with a mean age of 45.33±14.82 years. The value of Cronbach’s alpha indicated internal consistency; Split half reliability also indicated internal consistency within items of the scale. The KMO value of the Urdu version of SRMI was 0.63 (p<0.01), which showed the sampling adequacy of the scale. Bartlett’s test of sphericity indicates the suitability of the sample for factor analysis (p<0.01) (Table-II). Exploratory factors analysis structured two original factors of the Urdu version of the scale (Table-III). Convergent and discriminant validity of the Urdu version of SRMI indicated positive and negative correlations with similar and dissimilar constructs (Table-IV).

**Table-I:** Descriptive statistics of demographic variables.

Demographics	Categories	(f)	(%)	M	SD
Age				45.33	14.82
Gender	Male	136	68.0		
	Female	64	34.0		
Marital Status	Married	164	82.0		
	Single	32	16.0		
	Widow	4	2.0		
Family structure	Nuclear	155	77.5		
	Joint	45	22.5		
Stage of Disease	5	200	100		
Type of treatment	Hemodialysis	200	100		
Duration of diagnosis	Below 6 months	41	20.5		
	6-12 months	64	32		
	12-18months	8	4		
	18-24 months	30	15		
	24-30 months	1	.5		
	30-36 months	25	12.5		
	36-42 months	1	.5		
	42-48 months	13	6.5		
	48 months & above	17	8.5		

**Table-II:** Estimation of factor analysis and reliability analysis of Urdu version of Suicide risk scale of medical inpatient.

Reliability analysis		Factor Analysis	

KMO	χ^2^	Cronbach’s alpha (n=200)	Split half (n=200)
.69	251.88	.67	.68

**Table-III:** Exploratory factor analysis of the Urdu version of the suicide risk scale for medical inpatients (SRMI) by using the oblimin method (200).

No. of items	Items	Factor1	Fsctor2
Item no 2	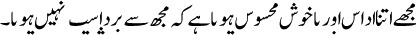	.80	
Item no 5	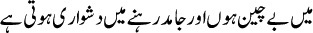	.71	
Item no 1	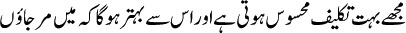	.69	
Item no 3	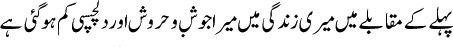	.52	
Item no 6	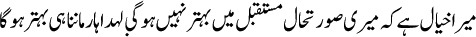		.83
Item no 4	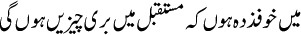		.75
Item no 7	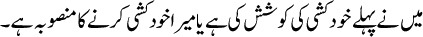		.73
Eigenvalues		34.74	20.62
% Of variance		34.74	20.60
Cumulative %		34.74	55.35

**Table-IV:** convergent and discriminant validity of the Urdu version of the Suicide Risk Scale for medical inpatients (SRMI).

Convergent validity		Discriminant validity
SRIM		
EFC	.24**	
ER	.39**	
PFC		-.08
IR		-.18**

***Note:***

** p<0.01, SRIM= Suicide risk scale for medical in patients, EFC= Emotional focused coping, ER= Extrinsic religiosity, PFC= Problem focused coping, IR= Intrinsic religiosity.

## DISCUSSION

The current study sought to translate and explore the psychometric properties of the Urdu version of SRMI among individuals with renal failure. In the first phase of the study, forward and backward translation procedures were completed by following five steps i.e., forward translation, committee approach, backward translation followed by committee approach, and pilot testing.^27^ In the second phase, the psychometric properties of the scale were established. Exploratory factor analysis (EFA) was done to verify the validity of the data. EFA structured two original factors, factor one includes four items i.e., one, two, three and five whereas factor two encompassed three items; items four, six and seven Kaiser-Meyer-Olkin (KMO) test is used to measure the proportion of variance in scale. The KMO index ranges from zero to one with 0.05 and the Bartlett`s Test of Sphericity should be significant (P<.05) considered to be suitable for factor analysis results of current study are in lined with previous literature.^29^-^32^ KMO value was .69 indicating sampling adequacy and substantial correlation. On the estimation of internal consistency, the partition of the test into two parts might be the only way to maintain content equivalence and if the parts are parallel, the Spearman-Brown formula is used to estimate the reliability of scores. Moreover, If the parts differ in their standard deviations but are equivalent, Cronbach’s alpha inappropriate.^30^-^32^ The value of both Cronbach’s alpha (.67) and split-half reliability (.68) showed internal consistency of the scale. In current data, SRMI significantly correlated with emotion focused coping scale and extrinsic religiosity subscale (convergent validity) and negatively correlated with problem focused coping and intrinsic religiosity subscale (discriminant validity), Cukroewicz et al, found similar results, according to them emotion-focused cop-ing is associated with suicidal ideation and hopelessness. Whereas, problem-focused coping lowers the suicide-related risks.^33^ Thus, the main results of current study showed that SRMI is a reliable and valid instrument for the screening of suicidal risk for medical inpatients. Suicide is a serious but preventable health issue if considered timely, the translated instrument is adding valuable knowledge to the medical literature and clinical fields. Patients having chronic illness experience suicidal ideation if their behavior and mental condition remain unaddressed it will eventually convert into suicide attempts therefore medical settings have to provide their best for suicide risk assessment and intervention.^28^ The beauty of this instrument is that it is translated into native Urdu language so that local population can easily understand and its psychometric properties were also established. It will facilitate health professionals to identify possible suicide risks and provide them intervention and support as soon as possible. Moreover, being brief it is used as a quick screening device it only takes 5-10 minutes for administration and scoring that, will help the professional to screen out possible risk of suicide its quick administration and makes it possible to assess patients who are in critical condition due to their physical illness.

### Limitation & future recommendation:

Though the main objective of the study has been attained, some limitations of the study need to be highlighted. In sample the ratio of male participants were greater as compared to female which limit the finding of study. Further studies are required along with longitudinal design and large more diverse sample. Moreover, Cross-cultural studies are suggested to understand the nature of the construct.

## CONCLUSION

Suicide risk for medical inpatients Urdu version is a brief seven items scale that is convenient for both participants and practitioners in terms of quick administration and scoring. The psychometric properties of the SRMI Urdu version were adequate. SRMI Urdu version can be used in Pakistan as well as across cultures. This scale assesses possible suicide risk in a medical setting so that medical aid or psychotherapy should be provided to improve their physical and mental health conjointly.
